# Intestinal linoleic acid contributes to the protective effects of *Akkermansia muciniphila* against *Listeria monocytogenes* infection in mice

**DOI:** 10.1002/imt2.196

**Published:** 2024-04-27

**Authors:** Tong Jin, Yingying Zhang, Yanpeng Yang, Yue Teng, Chunhong Yan, Zhongguo Shan, Jianghong Meng, Xiaodong Xia

**Affiliations:** ^1^ State Key Laboratory of Marine Food Processing and Safety Control, National Engineering Research Center of Seafood, School of Food Science and Technology Dalian Polytechnic University Dalian China; ^2^ Department of Food Safety, College of Food Science and Engineering Northwest A&F University Xianyang China; ^3^ Department of Food Science and Nutrition University of Maryland College Park Maryland USA

## Abstract

*Akkermansia muciniphila* pretreatment mitigated *Listeria monocytogenes* infection in mice. *A. muciniphila* improved gut microbiota disturbed by *L. monocytogenes* infection and significantly increased the level of intestinal linoleic acid in mice. Linoleic acid strengthened the intestinal epithelial barrier and reduced pathogen translocation partly by regulating NF‐κB/MLCK pathway in a GPR40‐dependent manner.

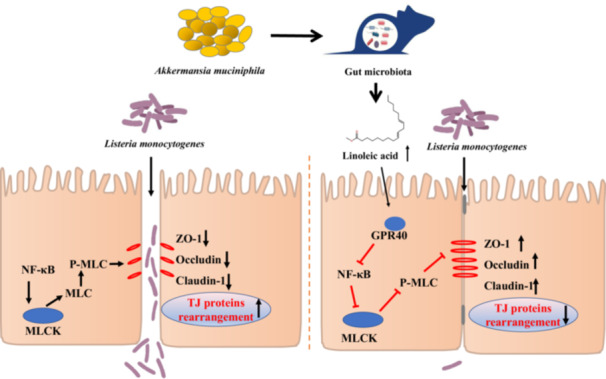

*Listeria monocytogenes* is a Gram‐positive bacterium that causes infections in humans ranging from mild gastroenteritis to severe systemic diseases such as septicemia and meningitis [1]. Populations susceptible to systemic infections include infants, pregnant women, the elderly, and immunocompromised individuals. *L. monocytogenes* is ubiquitous in nature and infects humans mostly via contaminated food products; therefore, it presents significant challenges to the food industry and public health [2]. Currently, there is a lack of an effective vaccine for listeriosis, and severe listerial infections are treated with various antibiotics. Considering the relatively high mortality rate of systemic listerial infection and the increasing concern about antibiotic resistance [3], it is highly imperative to explore novel, cost‐effective, and safe strategies to prevent listeriosis.

Commensal microbiota inhabiting the gastrointestinal tract have been demonstrated to confer protection against enteric infections through multiple mechanisms. They can bind receptors on intestinal cells and block pathogen‐epithelial interactions or directly inhibit enteric pathogens through nutrient competition or the production of bacteriocins or other antimicrobial molecules [4]. A consortium of commensals belonging to *Clostridiales* could confer resistance to listerial infection by directly inhibiting pathogen growth [5]. Gut commensals could also restrict infection by stimulating host defense systems such as the intestinal mucosal barrier and the immune cells underneath the gut epithelial cells [6]. *Enterococcus faecium* could enhance host barrier function by stimulating the secretion of the antimicrobial peptide RegIII to restrict *Salmonella* Typhimurium infection [7].

*Akkermansia muciniphila* is currently considered a promising next‐generation beneficial bacterium [8]. It has recently received increasing attention owing to its close association with metabolic diseases, such as obesity, type II diabetes, and nonalcoholic fatty liver disease [9]. Although the precise mechanisms underlying its beneficial functions are not fully elucidated, many of these pleiotropic effects are partially related to the bacteria's impact on mucin metabolism and gut barrier function [10]. Since crossing the intestinal barrier is an essential step for *L. monocytogenes* to disseminate into the circulatory system and cause systemic infection [1], it is intriguing to decipher whether *A. muciniphila* could exert a protective role against listerial infection. Although it has been recently reported that live *A. muciniphila* could reduce susceptibility to *L. monocytogenes* in mice fed a high‐fat diet mainly through modulating local inflammatory responses [11], whether and how live or pasteurized *A. muciniphila* impact *L. monocytogenes* infection in mice on a normal diet and the involvement of epithelial barrier have not been explored. Therefore, the aim of this study is to explore the impact of *A. muciniphila* supplementation on *L. monocytogenes* infection in normal diet‐fed mice and to identify potential mechanisms of action.

## RESULTS AND DISCUSSION

### **Live** *A. muciniphila* **attenuated** *L. monocytogenes* **infection in mice**

**Figure 1:**
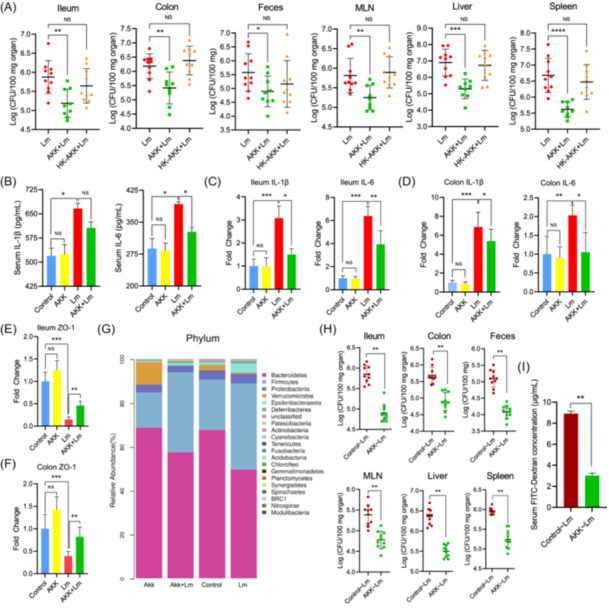
Live *Akkermansia muciniphila* alleviates listerial infection in mice through enhancing intestinal epithelial barrier and modulating gut microbiota. (A) Mice were administered by oral gavage with PBS (Lm group), live *A. muciniphila* (AKK) or heat‐killed *A. muciniphila* (HK‐AKK) for ten days, followed by oral inoculation of *L. monocytogenes* (Lm). *Listeria* loads in ileum, colon, feces, mesenteric lymph nodes (MLNs), liver and spleen were analyzed by plate counting (n = 10 for both Lm group and HK‐AKK+Lm group, n = 9 for AKK+Lm group due to death of one mouse owing to fighting). (B–D) *A. muciniphila* attenuated the inflammatory responses induced by *L. monocytogenes* in mice. (B) The concentrations of the pro‐inflammatory cytokines IL‐6 and IL‐1β in mouse serum. (C) Relative mRNA levels of inflammatory response‐related genes IL‐6 and IL‐1β in the murine ileum. (D) Relative mRNA levels of inflammation‐related genes IL‐6 and IL‐1β in the colon tissue of mice. (E and F) *A. muciniphila* ameliorates intestinal epithelial barrier dysfunction induced by *L. monocytogenes*, which was assessed by the relative mRNA levels of gene ZO‐1 in ileum (E) and colon (F) (*n* = 6 for each group). (G) Relative abundance of fecal microbiota at phylum level of mice from different groups. (H‐I) Gut microbiota from *A. muciniphila*‐treated mice reduced *Listeria* burdens in the murine organs and fecal samples, and attenuated *Listeria*‐induced intestinal barrier dysfunction (*n* = 10 for each group). Data are represented as the mean with SEM. *p < 0.05, **p < 0.01, ***p < 0.001, ****p < 0.0001. NS, no significant difference.

To determine whether *A. muciniphila* pretreatment influences the susceptibility of mice to *L. monocytogenes* infection, both live and heat‐killed bacteria were administered to mice by oral gavage for 10 days before infection. Live *A. muciniphila* mitigated animal weight loss induced by *L. monocytogenes* infection, whereas heat‐killed *A. muciniphila* showed little protective effects (Figure S1). Moreover, live *A. muciniphila* resulted in significantly reduced *L. monocytogenes* loads in the organs (ileum, colon, mesenteric lymph nodes (MLN), liver, spleen) and feces (*p* < 0.01) compared to mice in *L. monocytogenes* (Lm) group, whereas heat‐killed *A. muciniphila* did not exhibit similar protective effect (*p* > 0.05) (Figure 1A). Therefore, only live *A. muciniphila* was focused on in the following experiments.

In terms of inflammatory responses, live *A. muciniphila* pretreatment decreased the levels of interleukin (IL)‐6 and IL‐1β (Figure 1B) in serum induced by *L. monocytogenes*. In addition, real‐time quantitative polymerase chain reaction (RT‐qPCR) showed that *L. monocytogenes* upregulated the expression of IL‐6 and IL‐1β in the ileum (Figure 1C) and colon (Figure 1D), while *A. muciniphila* significantly downregulated the expression of these genes (*p* < 0.05). *A. muciniphila* also reduced *L. monocytogenes*‐induced upregulation levels of inflammation‐related genes tumor necrosis factor (TNF)‐α and inducible nitric oxide synthase (iNOS) in the ileum (Figure S2A,B) and colon (Figure S2C,D), Similarly, a recent report demonstrated that live *A. muciniphila* could mitigate *L. monocytogenes* infection mainly through modulating local inflammatory responses in high‐fat diet‐fed mice [11]. In the current study, we examined the effects of both live and pasteurized *A. muciniphila* on listerial infection in mice fed a normal diet and showed the protective role of live bacteria. The findings from these two studies could complement each other and demonstrate the broad contexts under which *A. muciniphila* could exert its anti‐listerial function.

### *A. muciniphila* alleviated *L. monocytogenes*‐induced dysfunction of the intestinal epithelial barrier

Since intestinal epithelial cells are the first line of defense against enteric pathogens such as *L. monocytogenes*, we first determined the integrity of the intestinal epithelial barrier of mice by detecting FITC‐dextran (fluorescein isothiocyanate‐dextran) permeability in vivo. *L. monocytogenes* infection increased the concentration of FITC‐dextran in serum (Figure S2E), while pretreatment with live *A. muciniphila* significantly reduced its level. The transcription levels of several genes related to the intestinal barrier in the ileum and colon were assessed with RT‐qPCR. Compared to the control mice, *L. monocytogenes* decreased the levels of Zona Occludens 1 (ZO‐1) (Figure 1E,F), Occludin and Claudin 1 (Figure S2F–I), whereas *A. muciniphila* pretreatment alleviated the downregulation of those genes caused by the pathogen (*p* < 0.05). These results indicate that *A. muciniphila* strengthen the intestinal epithelial barrier of mice partly by modulating the expression of junctional proteins, which is consistent with our previous study which showed live *A. muciniphila* could enhance the resistance of mice to *Salmonella* infection partly through strengthening the gut barrier [12].

### **Gut microbiota mediated protective effects of** *A. muciniphila* **against** *L. monocytogenes* **infection**

To investigate the effect of *A. muciniphila* pretreatment on the gut microbiota in *L. monocytogenes*‐infected mice, 16S rRNA amplicon sequencing analyses were performed. *L. monocytogenes* caused dysbiosis of the gut microbiota with a shift in bacterial composition and structure compared to the control group, and pretreatment with *A. muciniphila* significantly modulated the microbiome configuration in both normal mice and *L. monocytogenes*‐infected mice (Figure S3A,B). The composition and relative abundance of the gut microbiota are shown in Figure 1G. At the phylum level, the abundance of Verrucomicrobia significantly increased and the abundance of Firmicutes, Proteobacteria, Epsilonbacteraeota, Lactobacillus, and Eubacterium decreased significantly in the gut microbiota of *A. muciniphila*‐treated mice compared with the control mice (*p* < 0.05). However, *L. monocytogenes* infection induced a decreased abundance of Bacteroidetes and Verrucomicrobia in mice, while the abundance of Firmicutes and Epsilonbacteraeota increased significantly (*p* < 0.05). Compared with the Lm group, the abundance of Bacteroidetes increased, while the abundance of Firmicutes and Epsilonbacteraeota decreased in the AKK+Lm group (*p* < 0.05) (Figure S3C,D). In contrast, a high‐fat diet caused a lower abundance of *Bacteroidetes* and a higher abundance of Firmicutes, which predisposed mice to *L. monocytogenes* infection [13]. Keane et al. recently demonstrated that the protective effects of live *A. muciniphila* against *L. monocytogenes* infection in high‐fat diet‐fed mice may be not due to its effect on gut microbiota [11]. It is speculated that high‐fat diet may induce extensive alterations in microbiota in mice, which could not be reversed by short‐term *A. muciniphila* supplementation.

Fecal microbiota transplantation was performed to determine whether gut microbiota played an important role in the preventive function of *A. muciniphila* against *L. monocytogenes* infection in mice. Compared to mice receiving feces from mice in the control group, mice receiving feces from mice in the AKK group had reduced bacterial loads, accompanied by an improvement in intestinal epithelial barrier dysfunction (*p* < 0.01) (Figure 1H,I). These data showed that *A. muciniphila* pretreatment could modulate gut microbial community in mice fed a normal diet, which conferred resistance to listerial infection.

### *A. muciniphila* modulated linoleic acid levels in *L. monocytogenes*‐infected mice

**Figure 2:**
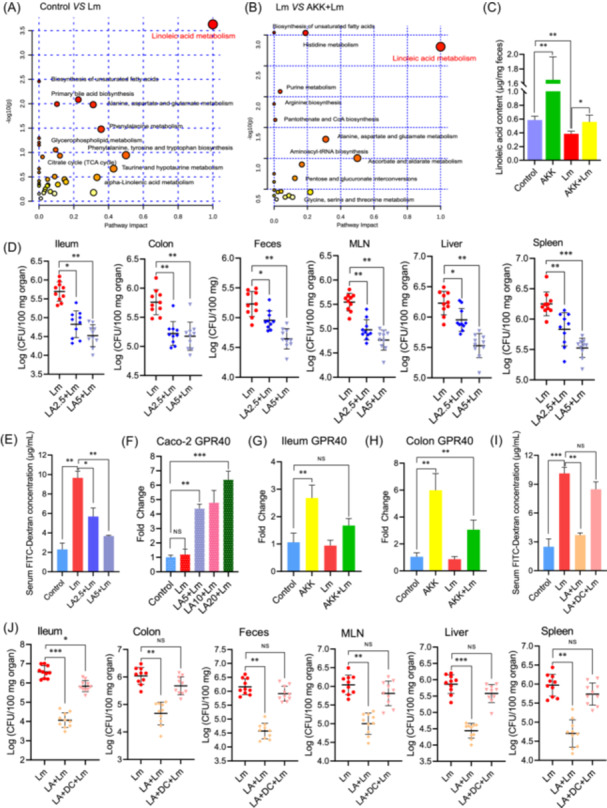
The gut metabolite linoleic acid (LA) attenuated *L. monocytogenes* infection by strengthens epithelial barrier in a GPR40‐dependent manner. Pathway analysis of metabolites in different groups (A: Control & Lm, B: Lm & AKK+Lm) are presented by bubble plots. (C) Quantitative measurements of LA in fecal samples from mice in different groups (*n* = 5 per group). (D) LA (5 or 2.5 g/kg) reduced *Listeria* burdens in the organs and fecal samples of mice. (E) The permeability of the intestinal epithelial barrier of mice treated with LA was evaluated by the concentration of FITC‐dextran in serum. (F) LA increased the relative mRNA level of GPR40 in Caco‐2 monolayers infected with *L. monocytogenes*. (G and H) *A. muciniphila* stimulated the mRNA levels of GPR40 in ileum (G) and colon (H) of mice. (I and J) Mice were treated with LA (5 g/kg) and a GPR40 inhibitor (DC260126, DC) (20 mg/kg). The concentration of FITC‐Dextran in serum (I, *n* = 10 per group), and *Listeria* loads in tissues and feces (J, *n* = 10 per group) were examined. *p < 0.05, **p < 0.01, ***p < 0.001. NS, no significant difference.

*A. muciniphila* has been reported to modulate circulatory conjugated bile acid levels to counteract severe fever with thrombocytopenia syndrome virus infection [14]. Further experiments were carried out to determine whether certain metabolites contributed to the protective effect of *A. muciniphila*. First, untargeted metabolomics analysis of mouse cecal contents was performed. PCA (Figure S4A) and heatmap (Figure S4B) analysis showed separation of the samples from different groups. As shown in Figure 2A,B and Figure S4C,D, linoleic acid metabolism pathway was significantly affected by AKK pretreatment during *Listeria* infection (*p* < 0.05). The concentrations of linoleic acid in fecal samples were also measured by HPLC‐MS (Figure 2C). Compared to the control mice, *L. monocytogenes* infection decreased linoleic acid levels, while *A. muciniphila* pretreatment increased its levels (*p* < 0.05). Linoleic acid in mice from the AKK+Lm group was recovered to almost the same level as that in the control group. These data showed that *A. muciniphila* supplementation effectively increased intestinal linoleic acid levels in mice. Keane et al. did not identify linoleic acid in their high‐fat diet model, which may be explained by the fact that they performed targeted analysis on short‐chain fatty acids in cecal contents and untargeted analysis of semi‐polar metabolites in the feces, which excluded the analysis of linoleic acid [11].

### Linoleic acid prevented *Listeria* infection in vivo and protected tight junctions in Caco‐2 monolayers in vitro

Since linoleic acid was enriched in the AKK‐treated group, it is intriguing to explore whether increased linoleic acid accounts for the protective effect of *A. muciniphila*. Oral pretreatment with linoleic acid significantly decreased *Listeria* bacterial loads in mice (*p* < 0.01) (Figure 2D) and attenuated damage to intestinal epithelial barrier (*p* < 0.01) (Figure 2E).

An in vitro Caco‐2 monolayer model was used to further explore the effects of linoleic acid on the host intestinal epithelial barrier and to decipher possible modes of action. Linoleic acid pretreatment decreased *L. monocytogenes* bacterial translocation through Caco‐2 monolayers (*p* < 0.05) (Figure S5A). Transepithelial electrical resistance and in vitro FITC‐dextran permeability test also indicated that linoleic acid mitigated *L. monocytogenes*‐induced loss of barrier function (Figure S5B,C). Taken together, these results demonstrated that linoleic acid could alleviate *L. monocytogenes* infection potentially through enhancing gut epithelial barrier.

### Linoleic acid exerts its protective effect in a GPR40‐dependent manner

We next tried to determine how linoleic acid could interact with epithelial cells to enhance the epithelial barrier. As linoleic acid is a strong endogenous agonist of the G protein‐coupled receptor 40 (GPR40) receptor [15], we wondered whether linoleic acid enhances the intestinal epithelial barrier partly by activating the GPR40 pathway. As shown in Figure 2F–H and Figure S5D, although *L. monocytogenes* did not alter GPR40 expression, linoleic acid treatment stimulated GPR40 activation in Caco‐2 cells (*p* < 0.01), which was consistent with increased transcriptional expression of GPR40 in ileum and colon of *A. muciniphila*‐treated mice (Figure 2G,H). GPR40 inhibitor (DC260126) blunted the protective effects of linoleic acid on *L. monocytogenes*‐induced damage to epithelial barrier of Caco‐2 monolayers, which was shown by its incapability to decrease the number of translocated bacteria (Figure S5E) and to recover barrier function (Figure S5F,G). Meanwhile, linoleic acid enhanced the epithelial barrier and suppressed the expression of inflammation‐related genes induced by *L. monocytogenes* (Figure S6). Linoleic acid did not reduce the apoptosis levels of Caco‐2 cells induced by *L. monocytogenes* (Figure S7), indicating that linoleic acid‐induced improvement in barrier function may be not due to a decrease in epithelial cell apoptosis. Consistent with in vitro results, DC260126 also nullified the protective role of linoleic acid against *L. monocytogenes* infection in mice (Figure 2I,J and Figure S8). It has been reported that *L. monocytogenes* could induce intestinal barrier dysfunction by activating NF‐κB and myosin light chain kinase (MLCK), resulting in cellular redistribution of epithelial junction proteins and bacterial translocation [16]. As shown in Figure S9A, linoleic acid rescued the reduction of ZO‐1, Occludin, Claudin‐1, and IκBα induced by *L. monocytogenes* in Caco‐2 monolayers. Linoleic acid also ameliorated the stimulation of MLCK and phosphorylation of MLC. Immunofluorescence staining showed linoleic acid downregulated nuclear translocation of NF‐κB p65 and relieved sub‐cellular redistribution of junctional proteins induced by *L. monocytogenes*. However, these changes induced by linoleic acid were absent with DC260126 treatment (Figure S9B). These data suggested that linoleic acid exerts its anti‐infective and barrier‐enhancing effect in a GPR40‐dependent manner.

## CONCLUSION

In summary, we demonstrated here that live *A. muciniphila* supplementation ameliorates *L. monocytogenes* infection partly through enhancing gut barrier function and increasing intestinal level of linoleic acid, which could modulate epithelial barrier integrity partly through the GPR40 pathway. Together, this work uncovers a novel pathway that could account for the barrier enhancement property of *A. muciniphila* and potentially facilitate the development of *A. muciniphila* or linoleic acid‐based prophylactic strategies against *L. monocytogenes* infection.

## AUTHOR CONTRIBUTIONS

Tong Jin and Xiaodong Xia conceived and designed the experiments. Tong Jin, Yingying Zhang, Yanpeng Yang, and Yue Teng performed the experiments. Chunhong Yan and Zhongguo Shan analyzed the data. Tong Jin and Xiaodong Xia wrote the manuscript. Jianghong Meng and Xiaodong Xia contributed to the manuscript revision. All the authors read and approved the final version of the manuscript.

## CONFLICT OF INTEREST STATEMENT

The authors declare no conflict of interest.

## ETHICS STATEMENT

The ethics application (No. DLPU2022082) was approved by the Animal Ethics Committee of Dalian Polytechnic University.

## Supporting information


**Figure S1**. Body weight of mice under various treatments.
**Figure S2**. *A. muciniphila* regulated the expression levels of genes related with inflammatory responses and tight junction in mice.
**Figure S3**. *A. muciniphila* modulated the gut microbiome in *L. monocytogenes*‐infected mice. 16S rRNA sequencing of cecal contents from different groups was carried out.
**Figure S4**. Metabolomic analysis of fecal samples of mice with different treatments.
**Figure S5**. GPR40 pathway is required for linoleic acid to exert protective effects against host intestinal epithelial barrier dysfunction induced by *L. monocytogenes in vitro*.
**Figure S6**. Linoleic acid regulated the expression levels of the inflammation‐related genes TNF‐α (A), IL‐6 (B), IL‐1β (C), PPARγ (D), TNFR1 (E) and TNFR2 (F) in Caco‐2 monolayers infected with *L. monocytogenes*.
**Figure S7**. Effects of linoleic acid and the GPR40 pathway on *L. monocytogenes*‐induced Caco‐2 apoptosis.
**Figure S8**. Body weight of mice under linoleic acid and GPR40 inhibitor treatments.
**Figure S9**. Linoleic acid influenced NF‐κB/MLCK activation and tight junction proteins redistribution induced by *L. monocytogenes* in Caco‐2 monolayers.

**Table S1**. Primer sequences for RT‐qPCR.

## Data Availability

The raw sequencing data generated in this study were stored in the National Center for Biotechnology Information (NCBI) Sequence Read Archive (SRA) database under BioProject accession number PRJNA867493. Supporting Information (methods, figures, tables, scripts, graphical abstract, slides, videos, Chinese translated version, and updated materials) may be found in the online DOI or iMeta Science http://www.imeta.science/.
