# Supplementary material for: Intestinal linoleic acid contributes to the protective effects of Akkermansia muciniphila against Listeria monocytogenes infection in mice
Source: Imeta. 2024 Apr 27;3(3):e196. doi: 10.1002/imt2.196 (PMC11183177; doi:10.1002/imt2.196)
Supplement: Supplementary file 1 — Figure S1. Body weight of mice under various treatments. Figure S2. A. muciniphila regulated the expression levels of genes related with inflammatory responses and tight junction in mice. Figure S3. A. muciniphila modulated the gut microbiome in L. monocytogenes‐infected mice. 16S rRNA sequencing of cecal contents from different groups was carried out. Figure S4. Metabolomic analysis of fecal samples of mice with different treatments. Figure S5. GPR40 pathway is required for linoleic acid to exert protective effects against host intestinal epithelial barrier dysfunction induced by L. monocytogenes in vitro. Figure S6. Linoleic acid regulated the expression levels of the inflammation‐related genes TNF‐α (A), IL‐6 (B), IL‐1β (C), PPARγ (D), TNFR1 (E) and TNFR2 (F) in Caco‐2 monolayers infected with L. monocytogenes. Figure S7. Effects of linoleic acid and the GPR40 pathway on L. monocytogenes‐induced Caco‐2 apoptosis. Figure S8. Body weight of mice under linoleic acid and GPR40 inhibitor treatments. Figure S9. Linoleic acid influenced NF‐κB/MLCK activation and tight junction proteins redistribution induced by L. monocytogenes in Caco‐2 monolayers. [file IMT2-3-e196-s001.docx]

**Supporting information to:** **Intestinal linoleic acid contributes to the protective effects of** ***Akkermansia* *muciniphila* against *Listeria monocytogenes* infection in mice**

**Running title:** *A. muciniphila* mitigates *L. monocytogenes* infection

**Authors:** Tong Jin^1,2^, Yingying Zhang,^2^ Yanpeng Yang^2^, Yue Teng^1^, Chunhong Yan^1^, Zhongguo Shan^2^, Jianghong Meng^3^, Xiaodong Xia^1,2*^

^1^State Key Laboratory of Marine Food Processing and Safety Control, National Engineering Research Center of Seafood, School of Food Science and Technology, Dalian Polytechnic University, Dalian, 116034, China;

^2^College of Food Science and Engineering, Northwest A&F University, Yangling, 712100, China

^3^Department of Food Science and Nutrition, University of Maryland, College Park, MD, 20742, the United States

^*^Correspondence: [foodscixiaodong@dlpu.edu.cn](mailto:foodscixiaodong@dlpu.edu.cn) (Xiaodong Xia)

**Materials and methods**

**Bacterial strains and culture conditions**

*A. muciniphila* (BAA-835) was purchased from the American Type Culture Collection (ATCC) (Gaithersburg, MD, USA). The *Listeria monocytogenes* (10403S InlA^m^) strain used in this study *L. monocytogenes* was grown in brain-heart infusion (BHI) broth (Landbridge, Beijing, China) at 37 °C overnight and resuspended in PBS at pH 7.4. *A. muciniphila* was cultured in BHI broth containing 0.5% mucin from porcine stomach (Sigma-Aldrich, St. Louis, MO, USA) and 0.03% L-cysteine (Sigma-Aldrich) in an anaerobic chamber at 37°C for 48 h and resuspended in anaerobic PBS at pH 7.4.

**Mice and infection**

Specific pathogen-free (SPF) male C57/BL6 mice (6 weeks old) were purchased from Xi’an Jiaotong University (SCXK 2013-003). The mice were fed a sterilized diet and water and housed in a controlled environment (21–23°C, 30–70% humidity, and 12/12 h light/dark cycle). Experiments were approved by the Animal Ethics Committee of Dalian Polytechnic University (DLPU2022082). After one week of acclimatization, the mice were randomly divided into six groups (10 mice per group) as follows: (1) control, (2) Lm, (3) AKK, (4) AKK+Lm, (5) (heat-killed) HK-AKK, and (6) HK-AKK+Lm. From Day 1 to Day 10, the mice from the control and Lm groups were fed PBS by oral gavage. Mice in the AKK and AKK+Lm groups were orally gavaged with 2×10^8^ CFU live *A. muciniphila*. Mice in the HK-AKK and HK-AKK+Lm groups were orally gavaged with pasteurized *A. muciniphila* (*A. muciniphila* suspensions were heated at 70°C for 30 min). On Day 11, the mice from the Lm, AKK+Lm and HK-AKK+Lm groups were administered with a single dose of 2×10^9^ CFU *L. monocytogenes* by oral gavage and then monitored for 3 days*,* and the mice in the control group received PBS. The mice in the AKK and HK-AKK groups were sacrificed without infection on day 11, and the mice in the control, Lm, AKK+Lm and HK-AKK+Lm groups were sacrificed on day 14.

**Intestinal permeability assay *in vivo***

Mice were orally gavaged with 4 kDa FITC Dextran (FD4, Sigma-Aldrich) to assess intestinal permeability. After 4 hours, the mice were anesthetized, blood was withdrawn, and serum was isolated. The fluorescence intensity of serum was measured with a Multi-Mode Microplate Reader (Spark, Tecan, Austria) at 485/528 nm (excitation/emission). The concentrations of FITC-Dextran in serum were calculated with the use of a standard curve.

**Bacterial enumeration**

Mouse tissues (ileum, colon, MLNs, liver, and spleen) and feces were collected aseptically and homogenized in cold sterile PBS containing 1% Triton X-100 (1:10, w/v). Serial dilutions were prepared and plated on *Listeria* Chromogenic Medium plates (HB7008, Hopebio, Qingdao, China) and incubated at 37°C for 24 h to determine the colony forming unit (cfu).

**Enzyme-linked immunosorbent assay (ELISA)**

Mouse serum was collected and analyzed via ELISA to measure the levels of Interleukin IL-6 and IL-1β according to the manufacturer’s guidelines of the kits (Xinle, Shanghai, China).

**RNA extraction and real-time quantitative polymerase chain reaction (RT-qPCR）**

Total RNA was extracted from colon, ileum, liver and spleen or Caco-2 cells using SteadyPure Universal RNA Extraction Kit II AG21022 (Accurate Biotechnology, Changsha, China) and then reverse transcribed to cDNA using Evo M-MLV RT Premix for qPCR (AG11706, Accurate Biotechnology) with the manufacturer’s instructions. Quantitative real-time PCR was performed with a SYBR^®^ Green Premix qPCR Kit (AG11701, Accurate Biotechnology) on a Bio-Rad iQ5 PCR system (Bio-Rad, Hercules, CA, USA). The primer sequences for RT-PCR are outlined in Table S1, using *GAPDH* as the housekeeping gene. All reactions were amplified in 45 cycles, and the relative mRNA levels were analyzed with Ct values.

**Microbial genomic DNA extraction and 16S rRNA gene sequencing**

Microbial genomic DNA was extracted from mouse cecal content samples (50 mg) using the PureLinkTM Microbiome DNA Purification kit (Thermo Fisher Scientific) [1]. The V3-V4 regions of bacterial 16S rRNA genes in each sample were amplified by PCR using the primers 338 F (5ʹ-ACTCCTACGGGAGGCAGCAG-3ʹ) and 806 R (5ʹ- GGACTACHVGGGTWTCTAAT-3ʹ). Purified V3-V4 amplicon libraries were pooled in equimolar amounts and paired-end sequenced (2 × 300) on an Illumina MiSeq platform (Illumina, San Diego, USA) with standard protocols. Raw reads were filtered using Prinseqlite v.0.20.4 by removing sequences with average quality scores below 20 (sequences shorter than 300 bp or those that were untrimmed). The resulting paired-end reads were overlapped using the merge program PEAR (v.0.9.10). Preprocessed sequences were assigned into operational taxonomic units (OTUs) using Usearch (version 7.0) with a 97% similarity threshold. The β-diversity was calculated based on principal coordinate analysis (PCoA) to identify microbial differences among samples. Statistical analyses of β-diversity used the vegan R package function Adonis and its default value of 999 permutations (https://cran.r-project.org/web/packages/vegan/index.html). The abundance of microbiota was calculated for differences between the mean proportions of each treatment. Significance in the classification of relative abundance between groups was determined using linear discriminant analysis effect size (LEfSe) analyses.

**Non-targeted metabolomics**

Gas chromatography-mass spectrometry (GC-MS)-based metabolomic analysis of cecal content was performed by Lianchuan-Bio (Hangzhou, China). The sample supernatant was transferred to a GC vial containing internal standards, dried under a gentle nitrogen stream and mixed with methoxyamine hydrochloride in pyridine. After vigorous vortexing, the resultant mixture was incubated at 37°C for 90 min, and BSTFA (with 1% TMCS) was added for derivatization. Then, an Agilent 7890A gas chromatography system coupled to an Agilent 5975C inert MSD system (Agilent Technologies, CA, USA) was used for sample analysis. The derivatives were separated utilizing an HP-5MS fused-silica capillary column. Then, helium was used as a carrier gas at a constant flow rate through the column. The samples were analyzed in a random sequence, and the peaks were aligned according to the m/z value and normalized migration time. The peak areas were calculated by normalizing against the internal standards, and the metabolites were identified by searching against the database based on the m/z value and normalized migration time. The differential metabolites were determined by the combination of the variable importance in the projection (VIP) value ( > 1) of the PLS-DA model and the *p* values < 0.05 from two-tailed Student’s t test on the normalized peak intensities. The structural identification of differential metabolites was performed using MetaboAnalyst 5.0, including retention time and mass spectra, Agilent Fiehn GC/MS Metabolomics RTL library, and Metabolome Database.

**Linoleic acid measurement**

The fecal linoleic acid level was measured by liquid chromatography (LC) as previously described with some modifications[2]. Mouse fecal samples were subjected to sequential solvent extraction by water and acetonitrile as described previously[3]. The analysis was performed on a Shimadzu LC-30AVP system (Shimadzu Co. Tokyo, Japan). Chromatographic separation was achieved by using a Zorbax SB-C18 column (4.6 mm × 50 mm, 1.8 μm, Agilent Technologies, Santa Clara, CA, USA) at 30°C and a 0.3 mL/min flow rate, and a 6 μL injection volume was used. Mobile phase A consisted of 0.1% (v/v) formic acid in water. Mobile phase B consisted of 0.1% (v/v) formic acid in acetonitrile. The gradient elution for the binary pump was linear gradient 40–45% B initially at 0–2 min; linear gradient 45–85% B at 2–30 min; 85% B at 30–35 min; linear gradient 85–95% B at 35–36 min; linear gradient 95–40% B at 36–37 min, and then 40% B at 37–40 min before the next injection. MS detection was performed on an API 5500 Qtrap triple quadrupole mass spectrometer from AB Sciex (Foster City, CA, US).

Detection was carried out in multiple reaction monitoring (MRM) in positive and negative ionization modes. The ion source parameters were as follows: ion spray voltage of − 4500 V, ion source temperature of 600°C, curtain gas (nitrogen) of 25 psi, ion source gas 1 of 45 psi, and ion source gas 2 of 40 psi. For qualitative and quantitative analysis, Analyst software 1.6.1 (AB Sciex, Foster City, CA, USA) was used.

**Fecal microbiota transplantation (FMT)**

FMT was carried out according to a previous study with minor modifications[4]. In brief, mice were administered an ANVM antibiotic cocktail (1 g/L ampicillin, 0.5 g/L vancomycin, 1 g/L neomycin, 1 g/L metronidazole in drinking water) (Abmole Bioscience Inc., Houston, USA) for 21 consecutive days. Fresh fecal pellets were collected from donor mice and homogenized in sterile PBS. Fecal suspensions were administered to recipient mice by oral gavage (200 μL/recipient) for 10 days. Then, after infection with *L.* *monocytogenes* for 3 days, we detected the bacterial loads in mouse organs and intestinal epithelial barrier permeability.

**Cell permeability assay**

Caco-2 cell permeability assays were conducted with Transwell inserts. Caco-2 cells were seeded in the upper chambers of 24-well transwell inserts (3.0-μm pore size polycarbonate filter; Corning, NY) for consistent 21 days until the monolayer was confluent for the tight junction structure. Cells were treated with serial concentrations of linoleic acid (20, 10, 5, 2.5 μg/mL) for 24 h. Then, *L.* *monocytogenes* suspensions (2×10^7^ CFU in 500 μL) were added to the upper chambers, and fresh FBS-free DMEM (800 µL) was added to the basal chambers. After incubation for 3 h, invading *L. monocytogenes* in basal chambers was counted by LB plating. The transepithelial electrical resistance (TEER) value was measured with a MillicellR ERS-2 meter (Millicells Voltmeter, Millipore, Massachusetts, United States). For analysis of FD4 flux, FD4 (1 mg/mL) was added to the upper chambers of transwell inserts. After 4 h of incubation, the medium in basal chambers was collected, and fluorescence was measured (Em: 485 nm; Ex: 520 nm; Spark, Tecan, Austria) with a Multi-Mode Microplate Reader (Spark, Tecan, Austria).

**RT-qPCR**

Caco-2 cells were treated with linoleic acid and infected with *Listeria* using the same methods used for Western blotting. Then, total RNA was extracted from Caco-2 cells according to the instructions of the SteadyPure Universal RNA Extraction Kit II AG21022 (Accurate Biotechnology). Quantitative real-time PCR was performed as previously described, and the relative mRNA levels of G protein-coupled receptor 40 (GPR40), tumor necrosis factor 1 (TNFR1) and tumor necrosis factor 2 (TNFR2) were detected (the primer sequences for RT-PCR are outlined in Table S1).

**Linoleic acid intervention in *L. monocytogenes* infected mice**

Briefly, the mice were pretreated with linoleic acid (Sigma) (2.5 g/kg and 5 g/kg body weight) for 10 days and then infected with *L.* *monocytogenes* as previously described. At 3 days postinfection, the pathogen loads in mouse organs and intestinal epithelial barrier permeability were determined.

To justify the role of GPR40 in mediating the protective effect of linoleic acid, the mice were pretreated with linoleic acid (Sigma) at a dose of 5 g/kg body weight) by oral gavage for 10 days and then infected with *L.* *monocytogenes* as previously described. DC260126 (20 mg/kg) was intraperitoneally injected into the mice immediately before the administration of linoleic acid. At 3 days postinfection, the pathogen loads in mouse organs and intestinal epithelial barrier permeability were determined.

***In vitro* effects of linoleic acid on epithelial barrier and involvement of GPR40 pathway**

As a measurement of GPR40 activation, intracellular Ca^2+^ levels were assessed by a fluorescent method. Caco-2 monolayers were incubated in 6-well plates and treated with DC260126 and linoleic acid. After infection with *L. monocytogenes*, the monolayers were incubated with Fluo-3 AM (Beyotime) and stained with Hoechst 33342. Intracellular Ca^2+^ activity was observed with a fluorescence microscope.

To justify whether GPR40 signaling pathway activation contributed to the protective effects of linoleic acid in *Listeria* infection, the GPR40 inhibitor DC260126 (25 μM) was used for verification. After treatment with DC260126, Caco-2 tight junction models were infected with *L. monocytogenes*, and bacterial translocation, TEER and FD4 flux were detected as described previously. In addition, Caco-2 cells were treated with an Annexin V-FITC Apoptosis Detection Kit (C1062, Beyotime, Shanghai, China), and cell apoptosis levels were measured with flow cytometry (CytoFLEX, Beckman, Brea, CA, USA).

**Western blotting and immunofluorescence**

Protein in cells with different above treatments was extracted using RIPA lysis buffer with proteinase and phosphatase inhibitors (Beyotime). Equal amounts of protein (30 mg) samples were separated with 10-15% SDS-polyacrylamide gel electrophoresis and then electrotransferred onto a 0.45 μm nitrocellulose membrane. After blocking with 5% BSA in TBST, the membranes were incubated with a primary antibody at 4°C overnight. Then, the membranes were washed with TBST three times and incubated with horseradish peroxidase-conjugated secondary antibodies at room temperature for 90 min. After washing with TBST, ECL reagents (Solarbio, Beijing, China) were applied, and the bands were captured and analyzed using a ChemiDoc XRS System (Bio-Rad, CA, United States).

The cellular distributions of Claudin-1, Occludin and NF-κB p65 were detected by immunofluorescence staining assays, Caco-2 cells were propagated on round glass coverslips within 24-well plates and incubated to tight junction monolayer formation, then incubated with linoleic acid and/or DC260126 and infected with *Listeria* (MOI = 100) using the same methods as Western Blot. After infection for 4 h, the monolayers were fixed and blocked with a buffer kit (Beyotime) following the instructions. After incubation with 1:100 dilutions of primary antibodies at 4°C overnight, Cy-3-labeled secondary antibodies were used at a 1:500 dilution at room temperature for 1 h incubation. Samples on the slides were washed with PBS and mounted onto microscope slides with antifade reagent with DAPI (Beyotime). Images of protein expression and distribution in Caco-2 monolayers were obtained with a fluorescence microscope (DMi8, Leica, Germany).

**Statistical analysis**

Statistical analyses were performed in SPSS software (Version 22.0; SPSS, Inc., Chicago, IL). The data are presented as the mean ± SEM. Differences between more than multiple groups were assessed using one-way ANOVA followed by Tukey’s multiple comparisons test, *p* value of less than 0.05 was considered statistically significant.

Supplemental Figures


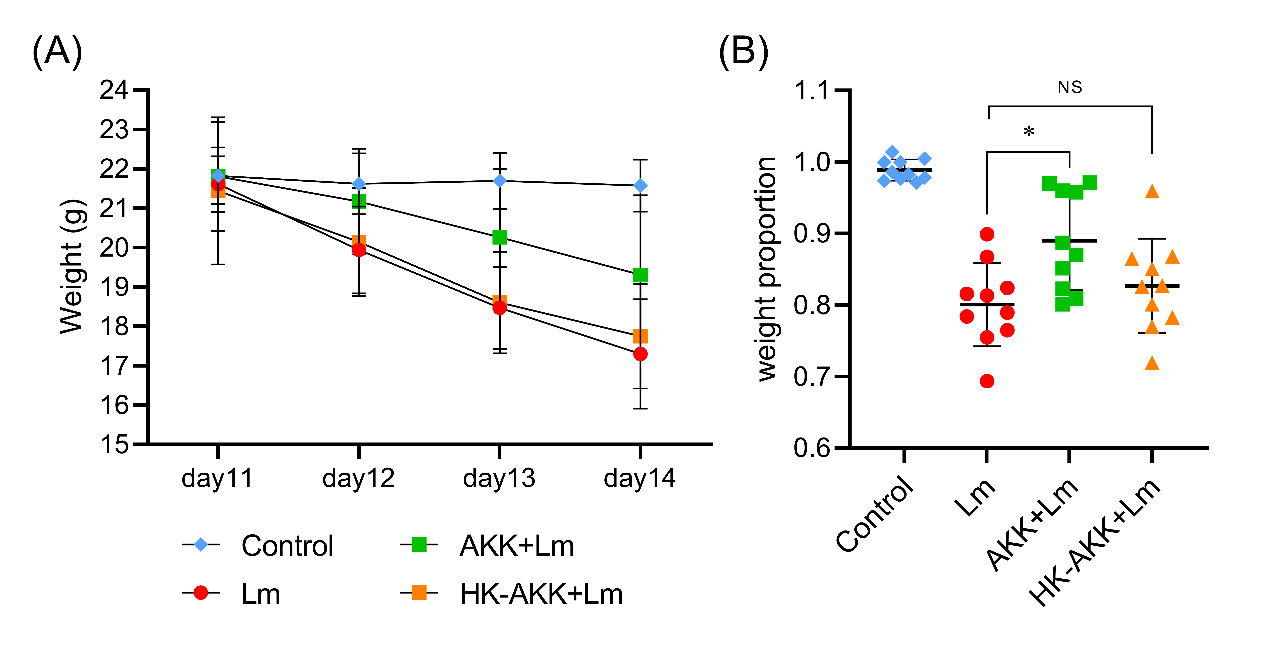


Figure S1 Body weight of mice under various treatments. (A) Changes in body weight (g) of mice with different treatments after *Listeria monocytogenes* infection (from day 11 to day 14). (B) The relative body weight of mice before sacrifice after *L. monocytogenes* infection (day 14) compared to the original body weight (day 11). Data are represented as the mean with SEM (n = 10 per group). **p* < 0.05. NS, no significant difference.


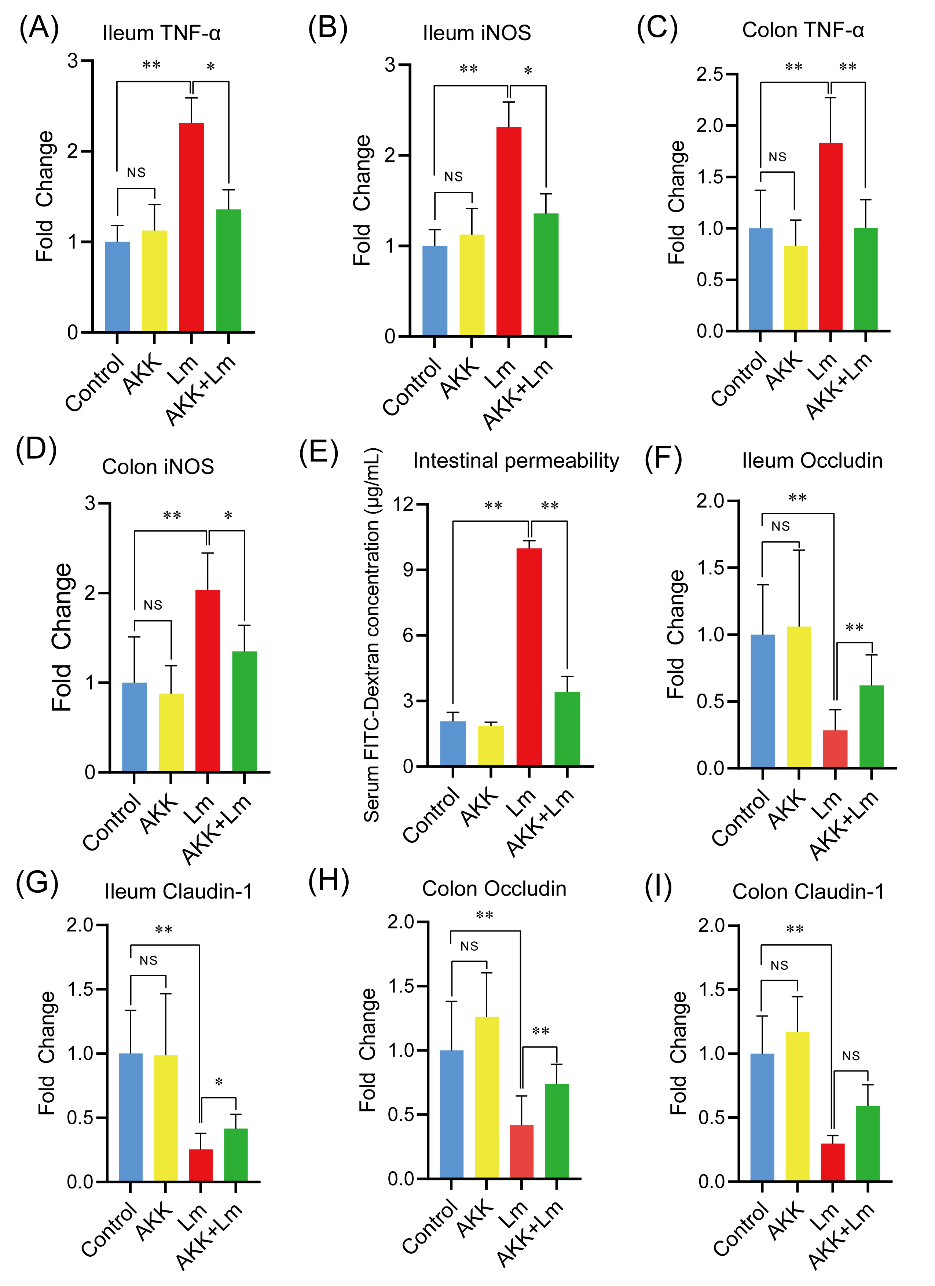


Figure S2 *A. muciniphila* regulated the expression levels of genes related with inflammatory responses and tight junction in mice. (A-D) Relative mRNA levels of inflammatory response-related genes TNF-α and iNOS in the murine ileum and colon. (E) The concentration of serum FITC-Dextran showed *A. muciniphila* mitigated the intestinal barrier dysfuction induced by *L. monocytogenes.* (F-I) Relative mRNA levels of Occludin and Claudin-1 in the ileum and colon tissue of mice. Data are represented as the mean with SEM (n = 6 per group), **p* < 0.05, ***p* < 0.01, NS, no significant difference.


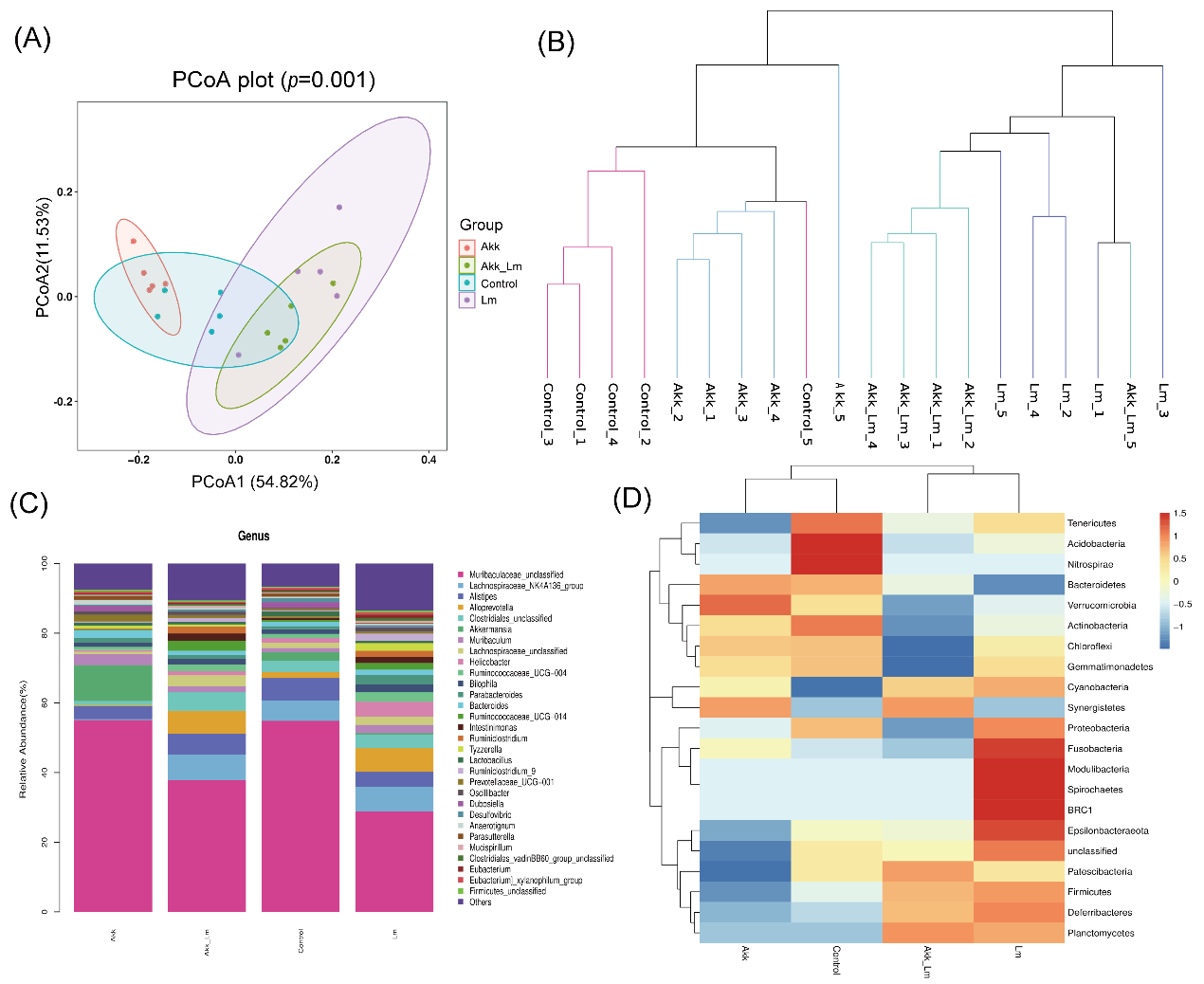


Figure S3 *A. muciniphila* modulated the gut microbiome in *L. monocytogenes*-infected mice. 16S rDNA sequencing of cecal contents from different groups was carried out. (A) Principal coordinates analysis (PCA) of the compositions of microbiota in the cecal contents of mice from different groups (n = 5 per group). (B) UPGMA sample clustering of gut microbiota from mice in each group. (C) Relative abundance of fecal microbiota at genus level of mice from different groups. (D) Heatmap demonstrating significantly altered bacterial taxa at family level in feces taken from mice in different groups.


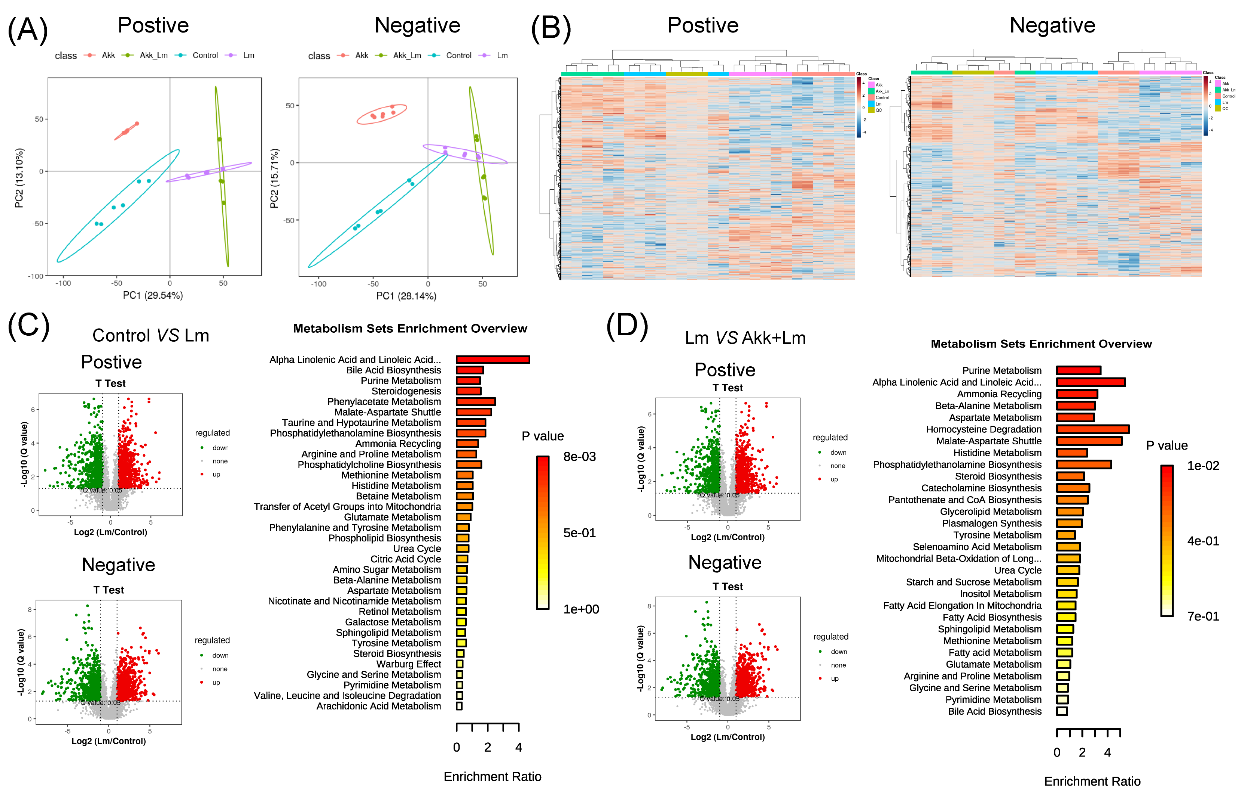


Figure S4 Metabolomic analysis of fecal samples of mice with different treatments. (A) Principal component analysis (PCA) of fecal metabolites (left: positive ion, right: negative ion) from different groups of mice (n = 5 per group). (B) Heatmap of fecal metabolites (left: positive ion, right: negative ion), which are significantly different (*p* < 0.05) in mice from different groups. (C) Volcano plots of significantly different ions of metabolites between mice in the control and Lm groups, and the top 25 differential metabolite pathways between the two groups are presented by bar charts. (D) Volcano plots of differential metabolites between mice in the Lm and AKK+Lm groups, and the top 25 differential metabolite pathways between the two groups are presented by bar charts. **p* < 0.05, ***p* < 0.01.


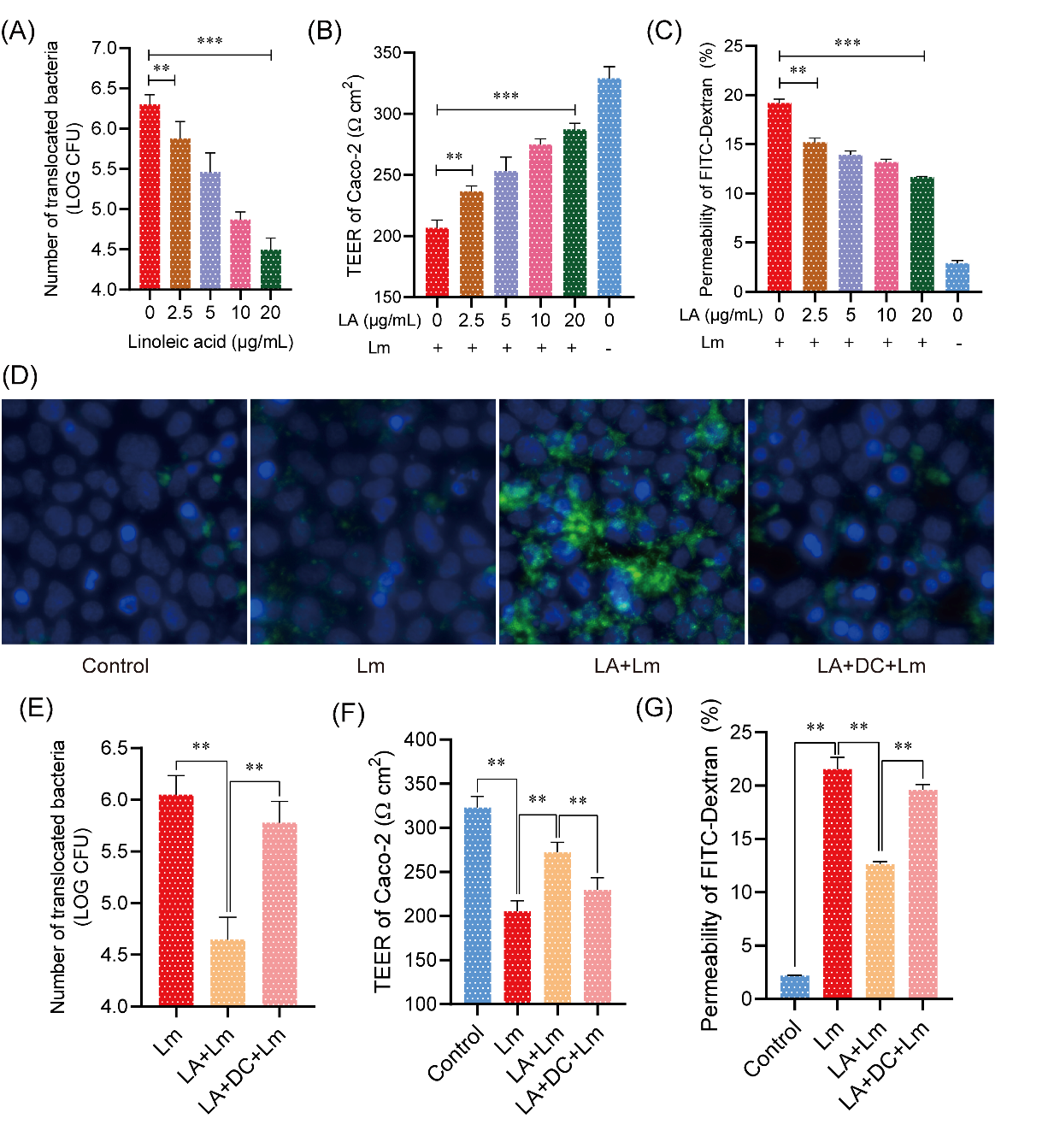


Figure S5 GPR40 pathway is required for linoleic acid to exert protective effects against host intestinal epithelial barrier dysfunction induced by *L. monocytogenes* *in vitro*. (A) The effects of linoleic acid on the number of *Listeria* translocated through the intestinal epithelial barrier model *in vitro*. (B&C) The TEER value (B) and 4 kDa FITC-Dextran permeability (C) of *L. monocytogenes-*infected Caco-2 monolayers pretreated with various concentrations of LA. (D) The intracellular calcium (Ca^2+^) activity (labeled with green fluorescence) in Caco-2 cells was examined by fluorescence staining to assess the activation status of GPR40. (E-G) Caco-2 monolayers were treated with LA and DC260126 (DC), and the number of *Listeria* translocated, the TEER value and FITC-Dextran (4 kDa) permeability of Caco-2 monolayers were determined. LA, linoleic acid. ***p* < 0.01,****p* < 0.001.


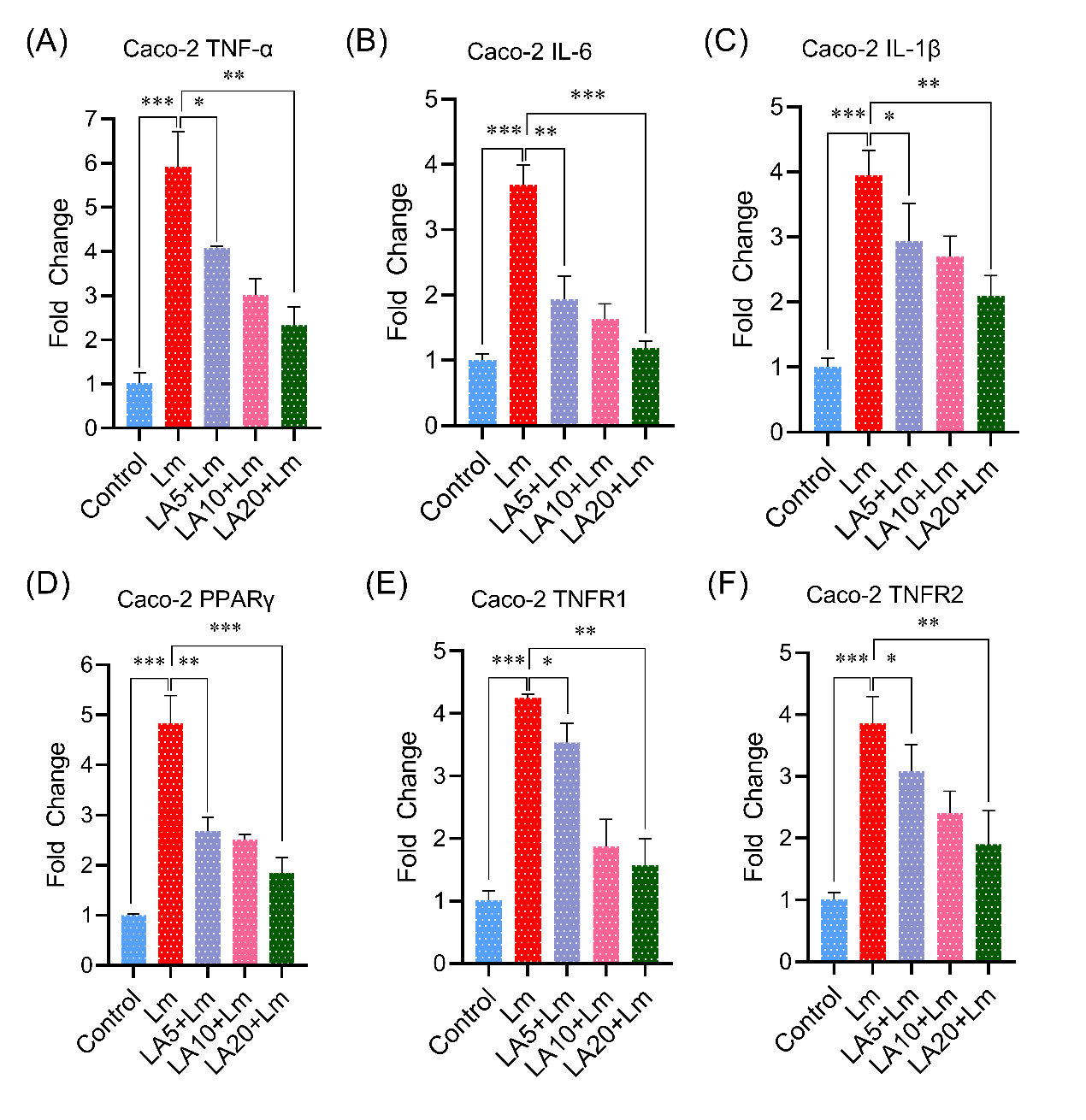


Figure S6 Linoleic acid regulated the expression levels of the inflammation-related genes TNF-α (A), IL-6 (B), IL-1β (C), PPARγ (D), TNFR1 (E) and TNFR2 (F) in Caco-2 monolayers infected with *L. monocytogenes*. Data are represented as the mean with SEM (n = 3). LA, linoleic acid. **p* < 0.05, ***p* < 0.01, ****p* < 0.001.


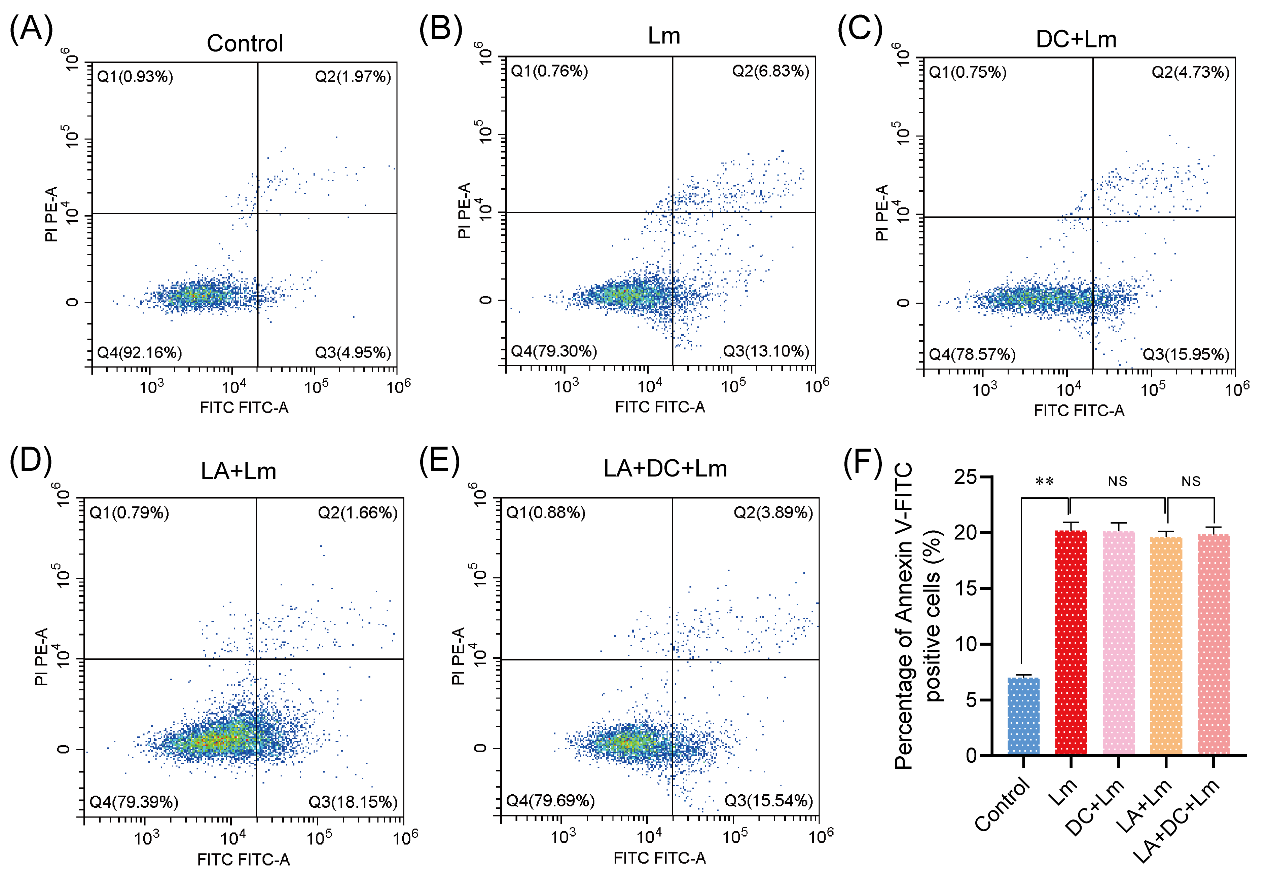


Figure S7 Effects of linoleic acid and the GPR40 pathway on *L. monocytogenes*-induced Caco-2 apoptosis. Caco-2 cells with different treatments (Control, Lm, LA+Lm, DC+Lm, LA+DC+Lm) were stained with Annexin V-FITC/PI and analyzed with a flow cytometer. The percentage of Annexin V-FITC-positive cells was calculated to assess the levels of Caco-2 apoptosis. Data are represented as the mean with SEM (n = 3 per group). LA, linoleic acid. ***p* < 0.01. NS, no significant difference.


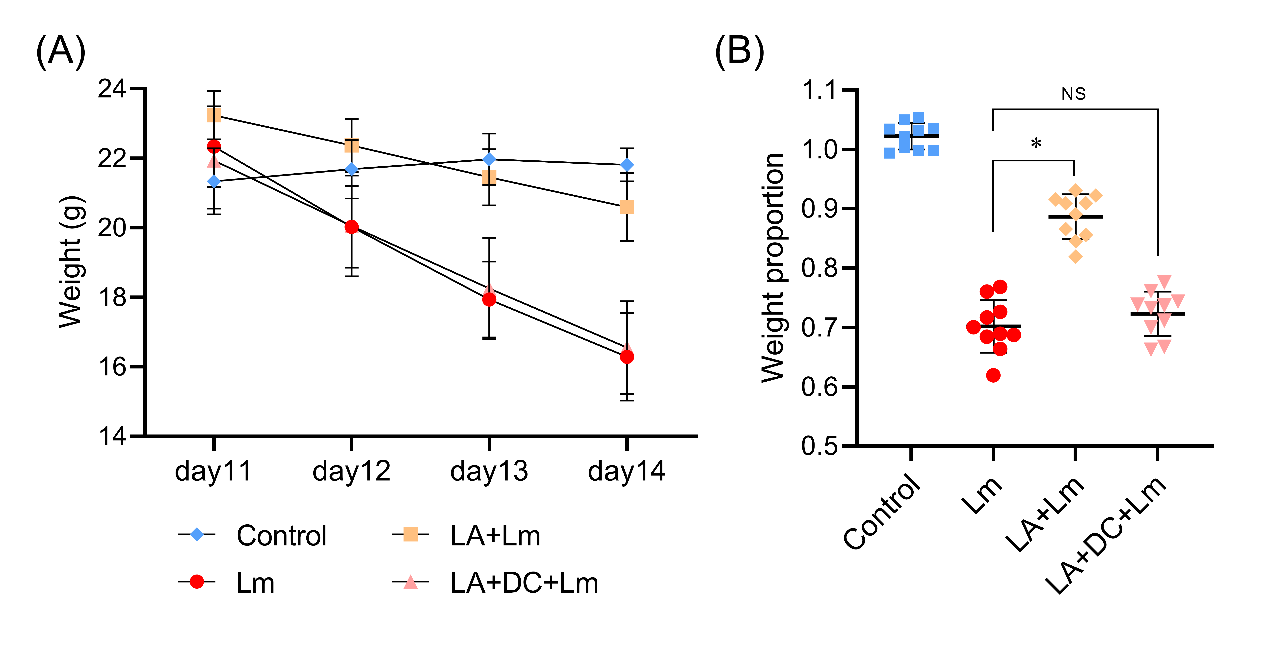


Figure S8 Body weight of mice under linoleic acid and GPR40 inhibitor treatments. (A) Changes in body weight (g) of mice with different treatments after *L. monocytogenes* infection (from day 11 to day 14). (B) The relative body weight of mice before sacrifice after *L. monocytogenes* infection (day 14) compared to the original body weight (day 11). Data are represented as the mean with SEM (n = 10 per group). LA, linoleic acid. **p* < 0.05. NS, no significant difference.


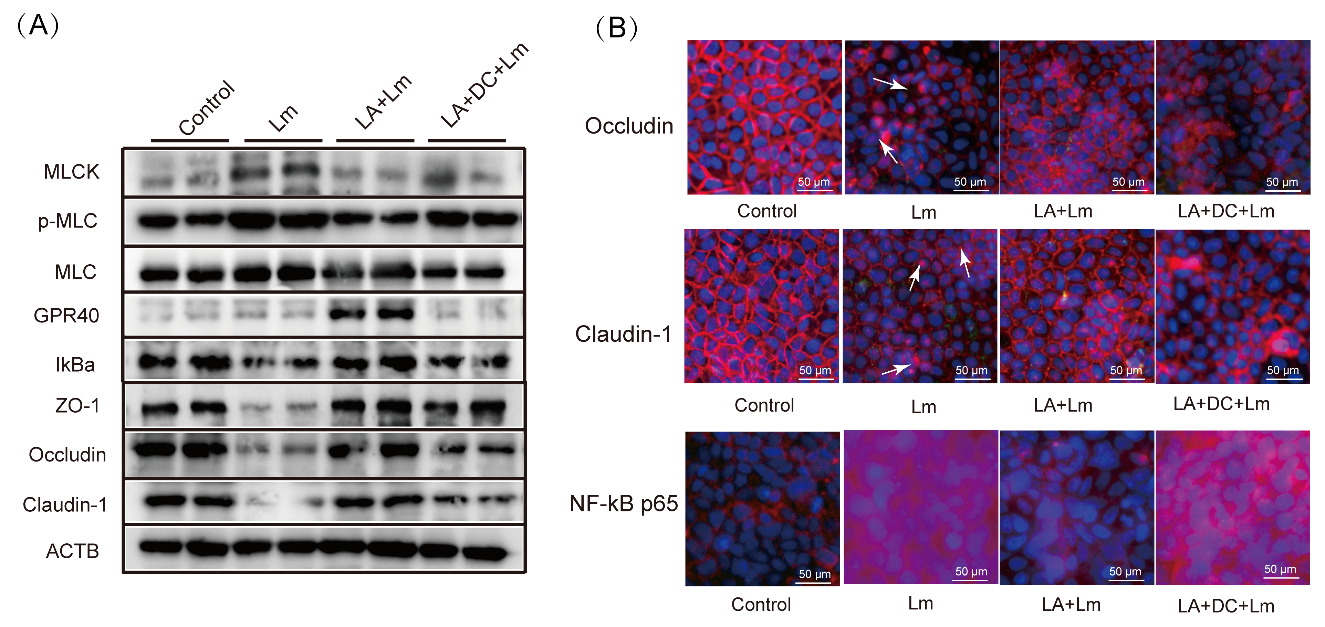


Figure S9 Linoleic acid influenced NF-κB/MLCK activation and tight junction proteins redistribution induced by *L. monocytogenes* in Caco-2 monolayers. (A) Representative western blots for proteins ZO-1, Occludin, Claudin-1, GPR40, MLCK, MLC, p-MLC, and IκBα in Caco-2 cells under different treatments (using ACTB as a control). (B) The expression and localization of proteins (Occlduin, Claudin-1 and NF-κB p65) (red fluorescence) on the Caco-2 monolayer (blue fluorescence for DAPI stained cell nucleus) infected with *L. monocytogenes* were analyzed with immunofluorescent staining. White arrows indicate sub-cellular redistribution of junctional proteins. LA, linoleic acid.

**REFERENCES**

1. Lu, [Xin-Yu](https://pubmed.ncbi.nlm.nih.gov/?term=Lu+XY&cauthor_id=33382357), [Bing Han](https://pubmed.ncbi.nlm.nih.gov/?term=Han+B&cauthor_id=33382357), [Xin Deng](https://pubmed.ncbi.nlm.nih.gov/?term=Deng+X&cauthor_id=33382357), [Si-Ying Deng](https://pubmed.ncbi.nlm.nih.gov/?term=Deng+SY&cauthor_id=33382357), [Yan-Yan Zhang](https://pubmed.ncbi.nlm.nih.gov/?term=Zhang+YY&cauthor_id=33382357), [Pei-Xin Shen](https://pubmed.ncbi.nlm.nih.gov/?term=Shen+PX&cauthor_id=33382357), *et al.* 2020. “Pomegranate peel extract ameliorates the severity of experimental autoimmune encephalomyelitis via modulation of gut microbiota.” *Gut Microbes* 12: 1857515. [https://doi.org/10.1080/19490976.2020.1857515](https://doi.org/10.1080/19490976.2020.1857515.)

2. Zhao, [Guan-Hua](https://pubmed.ncbi.nlm.nih.gov/?term=Zhao+GH&cauthor_id=34399536), [Yuan-Yuan Hu](https://pubmed.ncbi.nlm.nih.gov/?term=Hu+YY&cauthor_id=34399536), [Zhong-Yuan Liu](https://pubmed.ncbi.nlm.nih.gov/?term=Liu+ZY&cauthor_id=34399536), [Hong-Kai Xie](https://pubmed.ncbi.nlm.nih.gov/?term=Xie+HK&cauthor_id=34399536), [Min Zhang](https://pubmed.ncbi.nlm.nih.gov/?term=Zhang+M&cauthor_id=34399536), [Rui Zheng](https://pubmed.ncbi.nlm.nih.gov/?term=Zheng+R&cauthor_id=34399536), *et al.* 2021. “Simultaneous quantification of 24 aldehydes and ketones in oysters (*Crassostrea gigas*) with different thermal processing procedures by HPLC-electrospray tandem mass spectrometry.” *Food Research International* 147: 110559. <https://doi.org/10.1016/j.foodres.2021.110559>

3. Xu, [Wei,](https://pubmed.ncbi.nlm.nih.gov/?term=Xu+W&cauthor_id=25486321) [Deying Chen](https://pubmed.ncbi.nlm.nih.gov/?term=Chen+D&cauthor_id=25486321), [Nan Wang](https://pubmed.ncbi.nlm.nih.gov/?term=Wang+N&cauthor_id=25486321), [Ting Zhang](https://pubmed.ncbi.nlm.nih.gov/?term=Zhang+T&cauthor_id=25486321), [Ruokun Zhou](https://pubmed.ncbi.nlm.nih.gov/?term=Zhou+R&cauthor_id=25486321), [Tao Huan](https://pubmed.ncbi.nlm.nih.gov/?term=Huan+T&cauthor_id=25486321), *et al.* 2015. “Development of high-performance chemical isotope labeling LC-MS for profiling the human fecal metabolome.” *Analytical Chemistry* 87: 829-36. [https://doi.org/10.1021/ac503619q](https://doi.org/10.1016/j.foodres.2021.110559)

4. Jacobson, [Amanda,](https://pubmed.ncbi.nlm.nih.gov/?term=Jacobson+A&cauthor_id=30057174) [Lilian Lam](https://pubmed.ncbi.nlm.nih.gov/?term=Lam+L&cauthor_id=30057174), [Manohary Rajendram](https://pubmed.ncbi.nlm.nih.gov/?term=Rajendram+M&cauthor_id=30057174), [Fiona Tamburini](https://pubmed.ncbi.nlm.nih.gov/?term=Tamburini+F&cauthor_id=30057174), [Jared Honeycutt](https://pubmed.ncbi.nlm.nih.gov/?term=Honeycutt+J&cauthor_id=30057174), [Trung Pham](https://pubmed.ncbi.nlm.nih.gov/?term=Pham+T&cauthor_id=30057174), *et al.* 2018. “A gut commensal-produced metabolite mediates colonization resistance to *Salmonella* infection.” *Cell Host & Microbe* 24: 296-307. [https://doi.org/10.1016/j.chom.2018.07.002](https://doi.org/10.1016/j.chom.2018.07.002.)
